# Effect of a prospective payment method for health facilities on direct medical expenditures in a low-resource setting: a paired pre-post study

**DOI:** 10.1093/heapol/czaa039

**Published:** 2020-06-04

**Authors:** Ivlabèhiré Bertrand Meda, Seni Kouanda, Alexandre Dumont, Valéry Ridde

**Affiliations:** c1 Département Biomédical et Santé Publique, Institut de Recherche en Sciences de la Santé (IRSS/CNRST), BP 7192 Ouagadougou, Burkina Faso; c2 Département de Médecine Sociale et Préventive, École de Santé Publique de l’Université de Montréal (ESPUM), Montréal, Canada; c3 Institut Africain de Santé Publique (IASP), 12 BP 199 Ouagadougou, Burkina Faso; c4 IRD (French Institute for Research on Sustainable Development), CEPED (IRD-Université Paris Descartes), Universités Paris Sorbonne Cités, ERL INSERM SAGESUD, 45 rue des Saints Pères, 75006 Paris, France

**Keywords:** Health financing, health policy, costs, evaluation, exemption mechanisms, developing countries, policy evaluation

## Abstract

Almost all sub-Saharan countries have adopted cost-reduction policies to facilitate access to health care. However, several studies underline the reimbursement delays experienced by health facilities, which lead to deficient implementation of these policies. In April 2016, for its free care policy, Burkina Faso shifted from fee-for-service (FFS) paid retrospectively to FFS paid prospectively. This study tested the hypothesis that this new method of payment would be associated with an increase in direct medical expenditures (expenses covered by the policies) associated with deliveries. This paired pre-post study used data from two cross-sectional national surveys. Observations were paired according to the health facility and the type of delivery. We used a combined approach (state and household perspectives) to capture all direct medical expenses (delivery fees, drugs and supplies costs, paraclinical exam costs and hospitalization fees). A Wilcoxon signed-rank test was used to test the hypothesis that the 2016 distribution of direct medical expenditures was greater than that for 2014. A total of 279 pairs of normal deliveries, 66 dystocia deliveries and 48 caesareans were analysed. The direct medical expenditure medians were USD 4.97 [interquartile range (IQR): 4.30–6.02], 22.10 [IQR: 15.59–29.32] and 103.58 [IQR: 85.13–113.88] in 2014 vs USD 5.55 [IQR: 4.55–6.88], 23.90 [IQR: 17.55–48.81] and 141.54 [IQR: 104.10–172.02] in 2016 for normal, dystocia and caesarean deliveries, respectively. Except for dystocia (*P* = 0.128) and medical centres (*P* = 0.240), the 2016 direct medical expenditures were higher than the 2014 expenses, regardless of the type of delivery and level of care. The 2016 expenditures were higher than the 2014 expenditures, regardless of the components considered. In the context of cost-reduction policies in sub-Saharan countries, greater attention must be paid to the provider payment method and cost-control measures because these elements may generate an increase in medical expenditures, which threatens the sustainability of these policies.

**KEY MESSAGES**

- Direct medical expenditures (the portion of health expenses usually covered by cost-reduction policies in sub-Saharan countries) on deliveries and caesareans are higher when the payment of health facilities is made by prospective fee-for-service (FFS) than by retrospective FFS.
- This expense increase concerns all components (delivery fees, drugs and supplies costs, paraclinical exams costs and hospitalization fees).
- In the context of cost-reduction policies in sub-Saharan countries, greater attention must be paid to the provider payment method and cost-control measures because these elements may generate an increase in medical expenditures, which threatens the sustainability of these policies.

## Introduction

According to the World Bank, 37 of the 41 African countries that are currently using out-of-pocket payment at the point of service have adopted cost removal or reduction policies to increase access to care and reduce financial risk related to illness (Cotlear and Rosemberg, 2018). In the majority of cases, governments act as third-party payers, i.e. health facilities provide services to users with no financial contribution, and payments are borne by the government (Richard *et al.*, 2013).

The payment methods used by states (purchasers) vary across countries (Witter *et al.*, 2010, 2016; Richard *et al.*, 2013; Wang *et al.*, 2017; Andoh-Adjei *et al.*, 2018). Table 1 summarizes the payment methods used in some African countries with cost-reduction policies. Some (e.g. Ghana) use several methods, and others (Burkina Faso) have tried different methods successively.

**Table 1:** Provider payment methods used with cost-reduction policies in some African countries

Payment method	Definition	Payment rate determined	Payment made	Countries
Case-based	The health facility is paid a predetermined fixed rate for each treated case	Prospectively	Prospectively	Senegal (2005)
			Retrospectively	Benin (since April 2009), Mali (since January 2005), Burkina Faso (October 2006 to May 2010), Kenya (since June 2013)
Global budget	Payment to the health facility is fixed for all services delivered to all patients within a defined period	Prospectively	Prospectively	Morocco (since 2009)
Fee-for-service (FFS)	Payment is based on the individual components of health care		Retrospectively	Ghana (prescriptions)
				Burkina Faso (May 2010 to June 2016)
			Prospectively	Burkina Faso (since June 2016)
Capitation	A lump-sum payment is made for each patient enrolled for a defined period	Prospectively	Prospectively	Ghana (outpatient care)
Diagnosis-related groupings (case-based)	The health facility is paid one sum for all services delivered during one illness	Prospectively	Retrospectively	Ghana (inpatient care)

*Sources:* Witter *et al.* ( 2016), Richard *et al.* (2013), Ridde *et al.* (2011), Meda *et al.* (2019)and Wang *et al.* (2017).

The effects of these various payment methods on direct medical expenses (the portion of health expenditures usually covered by these policies), and therefore on the sustainability of these policies, have been minimally explored. A before-after controlled study conducted in three regions in Ghana showed that the shift from diagnosis-related groupings to a capitation payment method did not significantly reduce the expenditures for services provided (Andoh-Adjei *et al.*, 2018). In Burkina Faso, a pre-post study comparing the direct medical expenditures associated with deliveries during a case-based reimbursement period to those during a fee-for-service (FFS) reimbursement period in two health districts found that the direct expenditures significantly increased in some health facilities, decreased in others or remained unchanged in a third group. Overall, delivery expenses were significantly higher in one of the districts but not in another (Kiendrebeogo *et al.*, 2014). A recent review called for a cost evaluation of changes in payment methods, mainly in low-income countries (Yuan *et al.*, 2017). Indeed, depending on the payment methods used, health facilities have different incentives for containing costs (Park *et al.*, 2007; Langenbrunner *et al.*, 2009; Bodenheimer and Grumbach, 2016; Yuan *et al.*, 2017). An FFS payment method is considered to be associated with an increase in health expenses. For aggregated payment methods (capitation, global budget, case-based payment, etc.), health facilities risk losing money if they spend extra time and money on each patient, and therefore, they undertake cost-control measures.

In addition, incentives for cost containment may vary depending on whether the rate of payment is set prospectively or retrospectively, whether the payment is made prospectively or retrospectively or whether the payment is based on the inputs used or the outputs produced (Langenbrunner *et al.*, 2009). This variation may be especially true in low-income countries, where budgets are often inadequate. Some studies have shown that the reimbursement of health facilities according to cost-reduction policies in sub-Saharan Africa is often significantly delayed, leading to stockouts of drugs and consumables at health facilities (Witter *et al.*, 2008; Pariyo *et al.*, 2009; Ridde and Morestin, 2011; Ousseini and Kafando, 2012). In some countries, these policies are underfunded, and health facilities are commonly under-reimbursed (Ridde *et al.*, 2012b). We can hypothesize that these disruptive effects may unintentionally compel health facilities to use resources rationally and avoid bankruptcy.

From October 2006 to April 2016, Burkina Faso implemented a subsidy policy for deliveries and Emergency and Obstetric Care (EmONC) in the form of cost sharing between the government and users (Ridde *et al.*, 2011). Since April 2016, this policy has evolved into a free care policy, with 100% of direct medical expenses borne by the government (Meda *et al.*, 2019). The free care policy was accompanied by a switch in the method payment from retrospective FFS to prospective FFS, with the objective of avoiding delays in reimbursement.

However, by switching to a prospective FFS payment method, the government of Burkina Faso removed the risk that health facilities might lose money (as a result of delays in reimbursement or non-reimbursement) and therefore removed their financial incentive to control costs. This study was conducted to test the hypothesis that the payment of health facilities through a prospective FFS method would be associated with an increase in direct medical expenses compared with payment through a retrospective FFS payment method.

### Conceptual framework

In many low-income countries, households must pay out-of-pocket for health care at the point of service. These payments include direct medical expenses and direct non-medical expenses (Borghi *et al.*, 2003; Perkins et al., 2009). Direct medical expenses consist of delivery costs or service charges (registration fees, consultation fees, fees for care, cost of surgical intervention, etc.), costs for drugs and supplies (purchased inside or outside the health facility), costs for complementary exams (laboratory tests, radiology, ultrasound, etc.), hospitalization fees (bed stay costs) and the costs of transport by ambulance between health facilities in cases of evacuation to a referral hospital. These payments may be official or unauthorized. Direct non-medical expenses may include the costs of food for the patient and his or her accompanying relatives, the costs of accommodation for accompanying persons, the costs of transporting the patient and his or her accompanying persons to and from health facilities and gifts offered to health workers. In general, cost-reduction policies, which have been adopted by several sub-Saharan countries over the last two decades, cover direct medical expenses (Richard *et al.*, 2013).

Thus, under the subsidy policy in Burkina Faso, the Ministry of Health initially estimated a lump sum representing the medical expenses for each service covered, of which the government would bear 80% and the patient would bear 20%. In May 2010, the government replaced the case-based payment method with an actual cost-based reimbursement method (an FFS payment method), but patients continued to pay the same amount for each service covered, and the government reimbursed the health facility for the rest of the actual expenses. This change was motivated by the fact that the actual expenses were lower than the lump sum initially estimated by the Ministry of Health (Ridde *et al.*, 2011).

Under the free care policy, the Ministry of Health is supposed to cover the totality of these medical expenses (Meda *et al.*, 2019). However, studies have shown that under the subsidy policy, households pay more than the official flat rate (Ridde *et al.*, 2012a, 2013), and under the free care policy, households are still paying (Meda *et al.*, 2019). Our study addressed all of these direct medical expenses, regardless of whether they were paid by the household or the state (see Box 1). This approach allowed the comparison of expenditures between the two periods despite the differences in distribution between the state and households.

### Provider payment methods in cost-reduction policies in Burkina Faso

The two policies are described elsewhere (Ridde *et al.*, 2011; Meda *et al.*, 2019). In practice, under the subsidy policy, the health facility filled out a cost form with the different services provided and their prices and required the patient to bear the non-subsidized share. Health facilities reported monthly to the Health and Family Department of the Ministry of Health by specifying the services provided along with cost sheets. The department then checked for consistency in the claims expenditure prior to ordering the reimbursement of the health facility. Studies have shown that the delay in the reimbursement of health facilities could be as long as 12 months (Méda *et al.*, 2013).

Under the free care policy, at the beginning of each quarter, the Public Treasury transfers an amount equivalent to 3 months of the services covered by the free care policy into the account of each hospital and district. The transfer amounts were initially calculated by using the historical annual utilization of the different services for 2015 and estimating a cost for each service covered. Later, service utilization was estimated based on the activity of each health facility over the last 6 months. At the end of the quarter, the Public Treasury, at the request of the Ministry of Health, pays each hospital and district a cash amount for the three following months in consideration of the bank balance (positive or negative) for the previous quarter. Thus, health facilities permanently have funds in advance for the implementation of the free care policy. With the FFS method, payment is usually made retrospectively (Langenbrunner *et al.*, 2009), but now, the payment is made prospectively.

In addition to the internal control performed by departments of the Ministry of Health, the Ministry has established a contract with four international non-governmental organizations (NGOs) to ensure external control of the effectiveness of the free care policy for beneficiaries.

## Methods

### Setting

Burkina Faso is a low-income West African country. In 2016, the budget of the Ministry of Health accounted for 12.4% of the national budget, and over the last 5 years, it has maintained an apparently constant rate, varying from 12.4% to 12.7% (Ministère de la Santé, 2017). The Ministry of Health is responsible for paying the salaries of health personnel and managing investment expenditures. In addition, it funds the health districts and hospitals through a line-item budget (a given amount of money provided for specific line items) for equipment and operating expenditures. At the health facility level, resources from user fees and profits from selling drugs are used to pay operational expenses (the costs of cleaning products, refrigerator gas, etc.) and health centre workers’ salaries (security guards, housekeepers, pharmaceutical depot managers).

In terms of healthcare organizations, three types of health facilities may be distinguished: basic health centres, called Centres de Santé et de Promotion Sociale (CSPSs), medical centres (MCs) and hospitals (district, regional and university hospitals).

### Study design and data sources

We conducted a paired pre-post quasi-experimental study using data from two cross-national surveys conducted in 2014 (during the FFS reimbursement period) and 2016 (when the FFS was paid prospectively).

The two nationwide surveys were conducted by the Institut de Recherche en Sciences de la Santé (IRSS) using an identical methodology. This methodology is described elsewhere (Meda *et al.* 2018, 2019).

Briefly, the methodology used multistage stratified sampling, where the strata were the types of health facilities (CSPS, MCs and hospitals). The samples included all hospitals, MCs and randomly selected CSPSs in each region. Then, patients were randomly selected from each health facility. The number of patients varied according to the type of service and the type of health facility, with the number increasing with the level of care.

In both surveys, an exit interview was conducted with the patients to identify their education, occupation, marital status, age, parity, the type of service they used, the qualifications of care providers and direct medical expenses related to the service provided. The cost questionnaire identified all drugs and supplies used for care, including those purchased by the patient in the health facility’s pharmacy, from health workers or outside the health facility. The questionnaire also collected all paraclinical examinations and their prices, the length of hospitalization and its cost and the delivery fees. The information collected also included unauthorized payments to caregivers for care provided and payments for toilet use by the parturient.

The data accounted for 999 women from 546 health facilities in the 2014 survey and 593 women from 299 health facilities in the 2016 survey. We limited our study population to women who had a vaginal delivery or a caesarean section because only these services were included in both surveys.

One hundred sixty-one (161) health facilities were surveyed in both 2014 and 2016. We obtained 180 pairs of observations from 109 CSPSs, 4 MCs, 38 district hospitals, 8 regional hospitals and 2 university hospitals by pairing these health facilities by health facility and type of delivery. To increase the sample size, we then paired the observations surveyed for only 1 year according to the health region, type of health facility (CSPS, MC, district, regional or university hospital) and type of delivery, bringing the final sample to 393 pairs.

### Measure of direct medical expenditures

The items considered in the calculations of the direct medical expenses included delivery fees, drugs and supplies costs, the cost of paraclinical exams and hospitalization fees. For each component, we summed the amounts reported on the EmONC or free care claims expenditures and the amount the patients paid to the health facility, private pharmacy or private facility. Therefore, these expenses included the expenses borne by the government and the expenses borne by the patients (see Box 1). Informal payments for care were considered to be delivery fees, and payments for toilet use were included in the hospitalization fees since hospitalization should normally include access to the toilet. The cost of transport by ambulance between health facilities was not included because data on this expense were not collected in 2014. Direct medical expenditures obtained in local currency (XOF) were converted to US dollars using the average exchange rate from 2014 to 2016 (US$1 = 559.8183 XOF).

### Other study variables

The other variables selected for analysis included the sociodemographic characteristics of the parturients (age, parity), type of delivery, qualification of the service provider, health region and type of health facility. Parity was categorized as nulliparous (zero deliveries during health facility admission), multiparous (1–4 deliveries) and grand multiparous (at least 5 deliveries). The type of health facility was categorized as a CSPS, MC or hospital. The type of delivery included normal, normal with episiotomy, dystocia, dystocia with episiotomy and caesarean. The provider’s qualification was classified as physician, midwife, auxiliary midwife, nurse and surgeon assistant.

### Data cleaning and analysis

The data were analysed with the Stata software (Stata Corp) version 15.1. We first searched for and deleted duplicate observations. Then, direct medical expenditures were standardized by survey and type of delivery. Standardized expenses >3.29 were considered extreme values (Tabachnick and Fidell, 2013) and were deleted before proceeding to observation pairing.

The chi-square test (for categorical variables) and Student’s *t*-test (for continuous variables) were used to compare the characteristics of the two samples. To compare the direct medical expenditures between 2014 and 2016, we used the Wilcoxon signed-rank test because the distributions were asymmetrical. We compared total direct expenditures and then the distribution of expenses per component. The 2014 direct medical expenditures were also compared with the 2016 direct medical expenditures according to the type of care facility and health region.

To check that differences between the 2014 and 2016 medical expenditures were not due to differences in drug prices, we also compared the average prices and number of the main drugs and consumables used for a delivery between 2014 and 2016 by using Student’s *t*-test.

A sensitivity analysis was carried out using a sample of only 180 pairs from the same health facility surveyed in 2014 and 2016. The threshold was set at 0.05 (one-sided test) for all statistical tests.

### Ethical considerations

The two surveys complied with the agreement of the ethics committee for Health Research in Burkina Faso. The data did not include the names of the patients, and our report does not reveal the identity of the health facilities.

## Results

### Description of the sample

The sample was distributed in 260, 18, 74, 38 and 3 observation pairs in CSPSs, MCs and district, regional and university hospitals, respectively. These observation pairs comprised 227 (57.8%) normal deliveries, 52 (13.2%) normal deliveries with episiotomy, 38 (9.7%) dystocia deliveries, 28 (7.1%) dystocia deliveries with episiotomy and 48 (12.2%) caesareans. Twenty (5.1%) vs 104 (26.5%) patients paid for drugs because they were out of stock at the health facility pharmacy. This difference suggests that stockouts were, paradoxically, more frequent under prospective payment.

The demographic characteristics of the patients as well as the number of days of hospitalization did not differ significantly between the FFS reimbursement period and the prospective FFS payment period (Table 2).

**Table 2:** Characteristics of the samples in the retrospective FFS period and prospective FFS period

Characteristic	Retrospective FFS period	Prospective FFS period	*P*-value
	*n* = 393 (%)	*n* = 393 (%)	
Marital status (married)	381 (97.0)	380 (96.7)	0.84
Woman’s age (mean and SD)	24.9 (5.9)	25.2 (6.6)	0.50
Parity Nulliparous Multiparous Grand multiparous	*n* = 389 108 (27.7) 236 (60.7) 45 (11.6)	*n* = 393 131 (33.3) 213 (54.2) 49 (12.5)	0.17
Profession of provider Doctor Midwife Nurse Auxiliary midwife Surgical assistant	23 (6.1) 143 (38.0) 41 (10.9) 144 (38.3) 25 (6.7)	33 (8.4) 161 (41.0) 52 (13.2) 131 (33.3) 16 (4.1)	0.17
Hospital stay (mean and SD in days)	2.0 (1.3)	2.0 (2.2)	0.85

**Figure 1:**
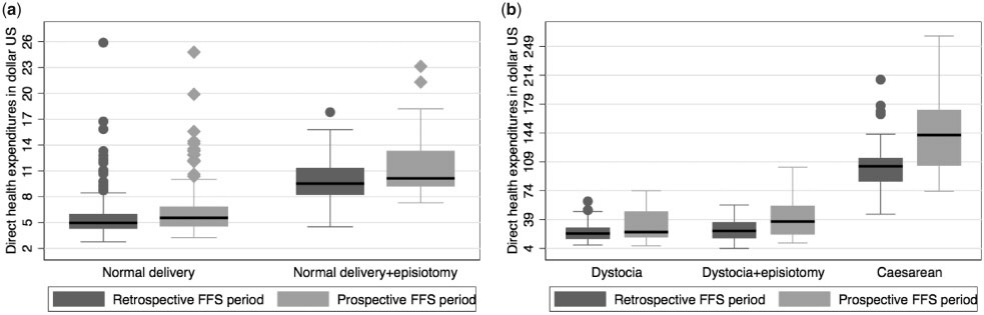
Distribution of direct medical expenditures between retrospective FFS and prospective FFS periods by type of delivery: (a) uncomplicated deliveries and (b) complicated deliveries.

Figure 1 shows the repartition of the direct medical expenditures per type of delivery before and after the health facility payment method changed.

### Comparison of the direct medical expenditures associated with deliveries

The results comparing the distributions of the total direct medical expenditures of the deliveries and the expenses according to the different components for the deliveries overall and by type are presented in Table 3.

**Table 3:** Comparison of direct medical expenses (median and interquartile range) in USD by type of delivery between the retrospective FFS and prospective FFS periods in Burkina Faso (primary analysis)

	Retrospective FFS period	Prospective FFS period	*P*-value
All deliveries	393	393	
Total expenses	6.78 (4.77–16.75)	7.72 (5.22–19.90)	<0.001
Delivery fees	1.34 (1.34–1.79)	1.52 (1.34–3.93)	<0.001
Drugs and supplies costs	4.45 (2.83–11.53)	5.24 (2.96–12.79)	<0.001
Paraclinical exams costs	0 (0–0)	0 (0–0)	<0.001
Hospitalization fees	0.71 (0.36–1.34)	0.89 (0.54–1.79)	<0.001
Normal delivery	227	227	
Total expenses	4.97 (4.30–6.02)	5.55 (4.55–6.88)	<0.001
Delivery fees	1.34 (1.34–1.34)	1.34 (1.34–1.43)	<0.001
Drugs and supplies costs	2.94 (2.36–3.79)	3.17 (2.51–4.36)	0.005
Paraclinical exams costs	0 (0–0)	0 (0–0)	0.13
Hospitalization fees	0.54 (0.36–0.89)	0.89 (0.36–1.07)	0.01
Normal delivery + episiotomy	52	52	
Total expenses	9.53 (8.23–11.35)	10.14 (9.21–13.32)	0.008
Delivery fees	1.34 (1.34–1.34)	1.65 (1.34–1.79)	<0.001
Drugs and supplies costs	7.69 (6.53–9.25)	7.94 (6.65–9.84)	0.06
Paraclinical exams costs	0 (0–0)	0 (0–0)	0.75
Hospitalization fees	0.54 (0.36–0.71)	0.80 (0.45–0.89)	0.003
Dystocia	38	38	
Total expenses	22.10 (15.59–29.32)	23.90 (17.55–48.81)	0.13
Delivery fees	2.01 (1.34–4.64)	3.57 (2.23–8.04)	<0.001
Drugs and supplies costs	12.81 (10.92–17.52)	14.55 (11.41–27.61)	0.21
Paraclinical exams costs	0 (0–7.15)	5.58 (0–12.50)	0.02
Hospitalization fees	1.07 (0.71–2.23)	1.79 (0.89–3.57)	0.13
Dystocia + episiotomy	28	28	
Total expenses	25.29 (16.29–35.84)	36.58 (20.85–55.78)	<0.001
Delivery fees	1.79 (1.34–3.39)	6.43 (3.35–9.02)	<0.001
Drugs and supplies costs	17.98 (11.25–23.14)	20.51 (14.60–32.69)	0.02
Paraclinical exams costs	0 (0–5.98)	6.03 (0–12.28)	<0.001
Hospitalization fees	1.07 (0.71–1.79)	1.79 (1.07–3.57)	0.001
Caesarean section	48	48	
Total expenses	103.58 (85.13–113.88)	141.54 (104.10–172.02)	<0.001
Delivery fees	17.86 (12.50–19.65)	20.10 (17.86–25.01)	<0.001
Drugs and supplies costs	68.26 (58.51–82.47)	105.19 (69.03–121.35)	<0.001
Paraclinical exams costs	6.88 (3.57–12.06)	9.51 (6.03–14.96)	<0.001
Hospitalisation fees	3.57 (1.79–5.36)	3.57 (2.41–5.36)	0.26

Average rate of exchange 2014–16: US$1 = 559.8183 XOF.

The total direct expenditures for all deliveries and for the different types of deliveries were generally higher after the payment method change, except for dystocia (*P* = 0.13).

According to the different expense components, the expenses after the health facility payment method changed were higher than the expenses prior to the change, regardless of the type of delivery, except for paraclinical exam costs for normal delivery (*P* = 0.13); drugs and supplies costs (*P* = 0.06) and paraclinical exam costs (*P* = 0.75) for normal delivery with episiotomy; caesarean hospitalization fees (*P* = 0.26); and drugs and supplies costs (*P* = 0.21) and hospitalization fees (*P* = 0.13) for dystocia deliveries.

The results of the sensitivity analysis (see Supplementary Table S1) were identical to the main analysis results, except for normal delivery with episiotomy, for which the distribution of medical expenditures did not change when the payment method changed (*P* = 0.50).

According to the type of health facility, the total expenses and component expenses for CSPSs and hospitals were higher after the payment method change than they were before the payment method change, except for paraclinical exams (*P* = 0.50) for CSPSs. The distributions of the total expenses and component expenses before and after the payment method change did not significantly differ at the statistical level for MCs. The comparative results of the distributions of the direct medical expenditures of the pre- and post-change periods per type of health facility are presented in Table 4.

**Table 4:** Comparison of direct medical expenses (median and interquartile range) in USD by type of facility between the retrospective FFS and prospective FFS periods in Burkina Faso

	Retrospective FFS period	Prospective FFS period	*P*-value
CSPS	260	260	
Total expenses	5.31 (4.50–7.54)	6.02 (4.62–8.53)	<0.001
Delivery fees	1.34 (1.34–1.34)	1.34 (1.34–1.61)	<0.001
Drugs and supplies costs	3.26 (2.52–5.71)	3.63 (2.61–6.03)	0.010
Paraclinical exams costs	0 (0–0)	0 (0–0)	0.50
Hospitalization fees	0.54 (0.36–0.89)	0.89 (0.36–0.89)	<0.001
Medical Centre	18	18	
Total expenses	5.94 (4.59–8.45)	7.15 (6.05–9.80)	0.24
Delivery fees	1.34 (1.34–1.34)	1.34 (1.34–1.61)	0.06
Drugs and supplies costs	4.01 (2.89–6.22)	4.51 (3.61–6.98)	0.12
Paraclinical exams costs	0 (0–0)	0 (0–0)	0.50
Hospitalization fees	0.54 (0.36–0.89)	0.89 (0.54–1.07)	0.055
Hospital	115	115	
Total expenses	40.37 (21.54–100.48)	63.98 (24.28–130.67)	<0.001
Delivery fees	6.43 (1.79–17.86)	11.61 (4.47–19.65)	<0.001
Drugs and supplies costs	24.08 (12.28–65.51)	32.86 (15.10–96.55)	<0.001
Paraclinical exams costs	4.47 (0–8.93)	7.15 (1.79–14.74)	<0.001
Hospitalization fees	1.79 (1.07–3.57)	2.68 (1.79–4.47)	0.02

Average rate of exchange 2014–16: US$1 = 559.8183 XOF.

CSPS, ‘Centre de santé et de promotion sociale’ (Basic Health centre).

Comparisons by health region show that the total direct medical expenditures post-payment method change were statistically higher than the total expenses before the payment method change in six regions (Cascades, Hauts-Bassins, Centre-East, North, Sahel and Southwest). The delivery fees were higher in 2016 than in 2014 in all but four regions (Centre-North, East, Sahel and Southwest). Four regions (Hauts-Bassins, Centre-East, North and Sahel) had expense distributions for drugs and supplies costs that were statistically higher in the post-change period than in the pre-change period. The East region was the only region where none of the health facilities’ expense distributions differed statistically between the pre- and post-payment method change periods, whereas for the Centre-East region, the expenses for all the components were significantly different.

**Figure 2:**
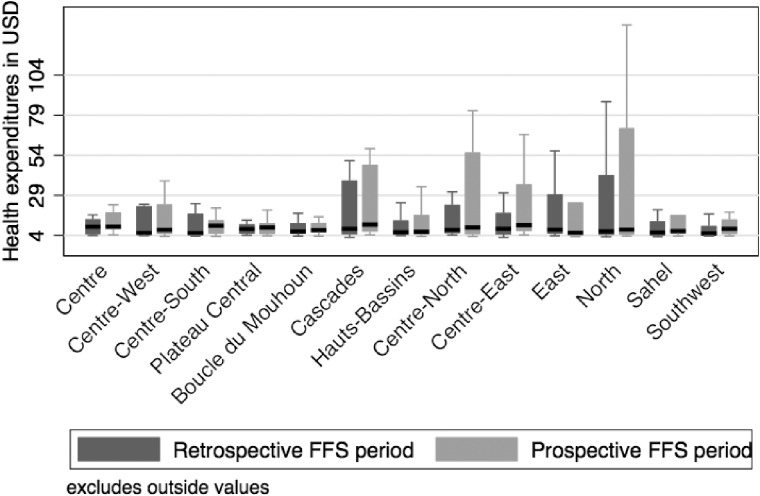
Distribution of direct medical expenditures between retrospective FFS and prospective FFS periods by sanitary region.

Figure 2 shows the distributions of total direct medical expenditures by health region before and after the payment method change. The results of the statistical comparison for the total expenses and the expenses by component are presented in Supplementary Table S2.

### Comparisons of the unit prices of drugs and consumables

From 2014 to 2016, the average unit prices of most of the different drugs did not significantly change. However, the average quantity of each drug and consumable used for one delivery was generally higher in 2016 than in 2014 (see Supplementary Table S3).

## Discussion

In general, the introduction of a cost-reduction policy in most African countries has been motivated by a political agenda or occurred under pressure from international organizations without careful preparation (Robert and Ridde, 2013; Ridde and Yaméogo, 2018). Policymakers have much less interest than researchers in the sustainability of these policies in general and, in particular, in the importance of the payment method for achieving policy objectives. This study is the third in sub-Saharan Africa to analyse the effects of a change in the health facility payment method on the medical expenses of services provided as part of a national cost-reduction policy to improve healthcare access (Kiendrebeogo *et al.*, 2014; Andoh-Adjei *et al.*, 2018).

In this study, the results confirm our hypothesis that paying health facilities prospectively is associated with an increase in direct medical expenditures (expenses covered by the policies). In fact, all components of expenses increased. In Burkina Faso, there are several incentives for health facilities to increase the direct medical expenditures for services provided: (1) profit from the selling of drugs and consumables, delivery fees and fees for different components is used to cover operating expenses; and (2) dividends calculated from health facility revenues (excluding the sale of drugs and consumables) are distributed quarterly to health personnel. Therefore, if the risk of losing money disappears (delay in reimbursement or non-reimbursement) and effective cost controls are not in place, health facilities can be encouraged to increase direct medical expenses.

Drugs and supplies costs were the component that saw the greatest increase, and the average quantity of each drug used per delivery also increased. According to the media, certain health workers overprescribe drugs and then sell the surplus in private clinics or to patients when medicines are out of stock. This observation is supported by the four NGOs, which documented 304 irregularities in 2017 concerning the free care policy, including unauthorized fees, informal payments, overcharging and fictitious patients. A previous publication based on the data of a 2016 survey (Meda *et al.*, 2019) confirmed that some health workers sold drugs to patients under the free care policy. One might think that this practice is encouraged by the fact that the actions taken against the alleged perpetrators were not sufficiently dissuasive. These actions were mainly limited to explanatory letters, warning letters or verbal warnings and transfer to another health facility. That sanctions are rarely enforced seems to be a universal problem. According to a recent review, the effectiveness of fraud prevention, detection and response for reducing these frauds and related spending is uncertain in low-income countries (Herrera *et al.*, 2017). In the case of Burkina Faso, this uncertainty raises the question of whether it makes sense to spend large sums of money for NGOs to document fraud if appropriate sanctions are never applied.

Unfortunately, this increase in drugs and supplies costs has not been associated with better availability of drugs because the results show that more patients paid for drugs outside of facilities in 2016 than in 2014. In fact, the establishment of the Generic Medicines and Medical Supplies Purchasing Centre (CAMEG), responsible for supplying public health facilities with essential generic drugs, experienced a deep governance crisis in 2016 and 2017 that disrupted drug supply.

The relevance of this study is its contribution to the ongoing debates regarding a better financing method for health facilities that will further actual adherence to cost reduction for care access policies for populations in sub-Saharan Africa and the sustainability of these policies.

Policymakers must draw more attention to the choice of payment system in general and to the payment method in particular because these elements should help to achieve the policy objectives (Langenbrunner *et al.*, 2009). The pre-financing mechanism that Burkina Faso introduced in 2016 may be a viable alternative financing system for promoting the effective adherence of health facilities to the free care policy. However, it does require control measures to ensure the efficient use of funding.

The method used to purchase services in health facilities must consider the local context and, in particular, the availability of cost-control measures. A possible alternative may be the strategic purchases suggested by some authors (Wang *et al.*, 2017; Paul *et al.*, 2018). The Ministry could adopt a flat-rate fee per case for the payment of health facility services. This flat rate would consider the quality of care offered by each health facility. Paying the health facilities with the best care providers more money would provide an incentive for the other health facilities. However, capping the amounts paid introduces a means of controlling costs. Such an approach may contribute to a cost-control system and therefore to the sustainability of a free care policy in this pre-financing context. However, the latter requires a good knowledge of the actual healthcare cost structure of the different services so that these services are paid at the minimum of their production costs. Unfortunately, studies on actual healthcare costs are still not widespread in sub-Saharan Africa (Perkins *et al.*, 2009; Dalaba *et al.*, 2013, 2015; Kalu-Umeh *et al.*, 2013; Meda *et al.*, 2018, 2019).

The study has methodological limitations that must be considered in the interpretation of the results.

First, the post-change survey was conducted only 5 months after the payment method changed, and we must ask ourselves if this time interval is sufficient for observing changes in the behaviour of different actors.

Second, the payment method change accompanied a cost-reduction policy change (from a partial subsidy of 80% to a free care policy), and it is legitimate to wonder whether this policy change had an impact on direct medical expenditures. Calculations of direct medical expenditures considered this policy change by including the share borne by patients to make pre- and post-change expenses comparable. We do not see any other way this change could have exerted an impact on direct medical expenditures.

Third, the calculation of direct medical expenditures included drugs purchased in private pharmacies when they were out of stock in the health facility pharmacy. This lack of availability frequently occurred during the free care policy period. However, the comparison of the average prices of the main drugs and consumables used for deliveries showed no significant difference between the pre- and post-payment method change periods. In addition, the increase in expenses concerned all components, not only drugs and consumables.

Fourth, the pairing did not consider the service providers’ qualifications, which may have an influence on drug prescriptions, complementary examinations and delivery fees. However, a comparison of the two samples showed no significant difference in the provider qualifications (*P* = 0.17).

Fifth, Figure 2 shows that a few regions (Centre-North, Centre-East and North) may have some cases with exceptionally high medical expenses and that this may have influenced the results of aggregated data at the national level. In this case, the results may reflect local factors (poor governance, practice patterns/provider culture) in a few specific regions rather than the effects of the change in payment method at the national level. However, 12 of the 13 regions showed higher medical expenses after the change in payment method. Of these, the differences were statistically significant for six regions, and for three regions, the differences were at the limit of statistical significance. In addition, we excluded extreme values by period and type of delivery prior to matching. Finally, the analyses used the median, which is more robust to extreme values than the mean. For all these reasons, we are reasonably confident that the results are generalizable at the national level.

Finally, in the subgroup comparisons, the number of patients treated at MCs was the lowest. A lack of statistical power may explain the non-significant expense increase at the MC level after the payment method change.

Despite the methodological weaknesses mentioned above, pairing increased the statistical power, and a sensitivity analysis showed robust results against a confounding bias due to potentially difference in health facility characteristics before and after the payment method change. Additionally, the study included all the health regions of the country and can be considered representative of the situation of Burkina Faso.

## Conclusion

Our results support the hypothesis that direct medical expenditures for deliveries would increase in association with the change in the payment method for health facilities. This increase covered the expenses for all items (delivery fees, drugs and supplies costs, paraclinical examinations costs and hospitalization fees) and affected all levels of care except for MCs.

The prospective payment of health facilities may regulate the problem of late reimbursement, which is considered one of the main barriers to health facilities’ adherence to cost-reduction policies in sub-Saharan Africa. However, greater attention must be paid to cost-control measures because the provider payment method may generate unexpected negative effects, such as an increase in medical expenditures, which threaten the sustainability of these policies.

## Supplementary data

Supplementary data are available at *Health Policy and Planning* online.

**Box 1 Working definition of direct medical expenses**

In this study, we were not interested in the reduction in out-of-pocket payment that could result from a shift from a subsidy policy to a free care policy (household perspective). We deliberately used a combined approach (payer and household perspectives) to capture direct medical expenses normally covered by cost-reduction policies that may be influenced by the change in payment method.

## Supplementary Material

czaa039_supplementary_data
