# Supplementary material for: Effect of a prospective payment method for health facilities on direct medical expenditures in a low-resource setting: a paired pre-post study
Source: Health Policy Plan. 2020 Jun 4;35(7):775–83. doi: 10.1093/heapol/czaa039 (PMC7487330; doi:10.1093/heapol/czaa039)
Supplement: czaa039_supplementary_data [file czaa039_supplementary_data.zip › Table S1.docx]

**Table S1.** Comparison of direct medical expenses (median and interquartile range) in USD by type of delivery between the retrospective fee-for-service (FFS) and prospective FFS periods in Burkina Faso (sensitivity analysis).

|  | Retrospective FFS period | Prospective FFS period | P-value |
| --- | --- | --- | --- |
| **All deliveries**  Total expenses    Delivery fees  Drugs and supplies costs  Paraclinical exams costs  Hospitalisation fees | **180**  10.26 (5.22-48.36)  1.34 (1.34-6.43)  7.74 (2.94-35.62)  0 (0-6.25)  0.89 (0.54-2.19) | **180**  12.15 (5.92-73.13)  2.19 (1.34-14.29)  8.62 (3.36-39.13)  0 (0-8.04)  1.07 (0.71-3.57) | <0.001  <0.001  <0.001  <0.001  0.01 |
| **Normal delivery**  Total expenses    Delivery fees  Drugs and supplies costs  Paraclinical exams costs  Hospitalisation fees | **84**  5.06 (4.35-5.94)  1.34 (1.34-1.34)  2.91 (2.37-3.71)  0 (0-0)  0.71 (0.36-0.89) | **84**  5.73 (4.60-7.04)  1.34 (1.34-1.38)  3.20 (2.51-4.18)  0 (0-0)  0.89 (0.54-1.07) | 0.02  0.001  0.03  0.25  0.13 |
| **Normal delivery + episiotomy**  Total expenses  Delivery fees  Drugs and supplies costs  Paraclinical exams costs  Hospitalisation fees | **11**  9.68 (8.15-10.24)  1.34 (1.25-1.34)  7.54 (5.74-8.23)  0 (0-0)  0.71 (0.36-0.89) | **11**  10.42 (9.07-11.52)  1.34 (1.34-1.79)  7.96 (6.42-9.11)  0 (0-0)  0.54 (0.36-0.71) | 0.50  0.02  0.50  1.0  0.89 |

Average rate of exchange 2014-2016: US$1 = 559.8183 XOF.

**Table S1 (Continued).** Comparison of direct medical expenses (median and interquartile range) in USD by type of delivery between retrospective fee-for-service (FFS) and prospective FFS periods in Burkina Faso (sensitivity analysis).

|  | Retrospective FFS period | Prospective FFS period | P-value |
| --- | --- | --- | --- |
| **Dystocia**  Total expenses    Delivery fees  Drugs and supplies costs  Paraclinical exams costs  Hospitalisation fees | **28**  24.12 (18.56-35.83)  2.50 (1.79-6.43)  13.23 (11.34-21.03)  2.23 (0-7.59)  1.79 (0.98-3.48) | **28**  40.39 (20.83-54.01)  4.47 (2.68-10.00)  17.78 (12.27-29.34)  6.03 (0-14.74)  1.79 (0.98-3.57) | 0.09  0.001  0.29  0.03  0.41 |
| **Dystocia + episiotomy**  Total expenses    Delivery fees  Drugs and supplies costs  Paraclinical exams costs  Hospitalisation fees | **15**  32.29 (20.49-41.26)  1.88 (1.34-4.64)  21.22 (13.40-28.57)  5.36 (0-12.06)  0.89 (0.71-2.23) | **15**  50.36 (34.36-68.99)  7.32 (6.80-13.58)  28.70 (17.39-36.59)  8.04 (5.81-17.42)  1.79 (1.79-3.57) | <0.001  0.002  0.06  0.007  0.006 |
| **Caesarean section**  Total expenses  Delivery fees  Drugs and supplies costs  Paraclinical exams costs  Hospitalisation fees | **42**  104.49 (84.21-114.16)  17.86 (12.50-21.44)  68.26 (58.26-82.04)  7.15 (3.93-12.50)  3.57 (1.79-5.36) | **42**  146.06 (104.65-176.54)  20.63 (17.86-25.01)  107.19 (66.89-121.98)  10.05 (6.25-16.08)  4.47 (3.57-6.25) | <0.001  <0.001  <0.001  0.002  0.16 |

Average rate of exchange 2014-2016: US$1 = 559.8183 XOF
