# Supplementary material for: Effect of a prospective payment method for health facilities on direct medical expenditures in a low-resource setting: a paired pre-post study
Source: Health Policy Plan. 2020 Jun 4;35(7):775–83. doi: 10.1093/heapol/czaa039 (PMC7487330; doi:10.1093/heapol/czaa039)
Supplement: czaa039_supplementary_data [file czaa039_supplementary_data.zip › Table S2.docx]

**Table S2.** Comparison of direct medical expenses (median and interquartile range) in USD by region between the retrospective fee-for-service (FFS) and prospective FFS periods in Burkina Faso.

|  | Retrospective FFS period | Prospective FFS period | P-value |
| --- | --- | --- | --- |
| **Centre**  Total expenses  Delivery fees  Drugs and supplies costs  Paraclinical exams costs  Hospitalization fees | **n=17**  9.39 (4.79-14.0)  1.34 (1.34-1.34)  7.15 (2.82-12.39)  0 (0-0)  0.89 (0.27-0.89) | **n=17**  9.49 (7.87-18.25)  1.79 (1.61-4.69)  7.26 (4.51-12.89)  0 (0-0)  0.89 (0.89-0.89) | 0.17  0.02  0.17  0.06  0.29 |
| **Centre-West**  Total expenses  Delivery fees  Drugs and supplies costs  Paraclinical exams costs  Hospitalization fees | **n=31**  5.51 (4.89-22.40)  1.34 (1.34-2.50)  3.47 (2.83-11.30)  0 (0-7.15)  0.71 (0.36-1.79) | **n=31**  7.45 (5.03-23.32)  1.34 (1.34-6.43)  5.31 (2.97-15.10)  0 (0-0)  0.54 (0.36-1.79) | 0.24  0.003  0.14  0.75  0.99 |
| **Centre-South**  Total expenses  Delivery fees  Drugs and supplies costs  Paraclinical exams costs  Hospitalization fees | **n=22**  5.43 (4.51-17.57)  1.34 (1.07-3.57)  3.83 (2.88-11.14)  0 (0-0)  0.54 (0.54-0.71) | **n=22**  10.08 (5.22-13.46)  1.56 (1.25-3.12)  8.10 (2.45-11.16)  0 (0-0)  0.54 (0.36-0.71) | 0.06  0.01  0.42  0.03  0.23 |
| **Central Plateau**  Total expenses  Delivery fees  Drugs and supplies costs  Paraclinical exams costs  Hospitalization fees | **n=46**  8.02 (5.09-11.12)  1.34 (1.34-1.34)  6.24 (3.09-9.19)  0 (0-0)  0.54 (0.36-0.54) | **n=46**  8.94 (4.97-11.64)  1.34 (1.34-2.23)  6.55 (2.98-8.82)  0 (0-0)  0.54 (0.36-0.89) | 0.052  0.003  0.28  0.004  0.03 |
| **Boucle du Mouhoun**  Total expenses  Delivery fees  Drugs and supplies costs  Paraclinical exams costs  Hospitalization fees | **n=54**  6.47 (5.44-11.61)  1.34 (1.34-1.34)  4.36 (3.40-9.74)  0 (0-0)  0.85 (0.54-1.07) | **n=54**  7.23 (5.99-11.76)  1.34 (1.34-1.79)  4.69 (3.58-6.95)  0 (0-0)  1.07 (0.89-1.07) | 0.21  0.004  0.39  0.06  0.008 |
| **Cascades**  Total expenses  Delivery fees  Drugs and supplies costs  Paraclinical exams costs  Hospitalization fees | **n=20**  8.16 (4.71-38.29)  1.61 (0.89-6.43)  6.32 (2.39-25.67)  0 (0-0)  0.89 (0.71-1.79) | **n=20**  10.74 (6.32-48.13)  2.23 (1.34-10.72)  7.61 (3.74-26.82)  0 (0-5.81)  0.89 (0.89-1.79) | 0.02  0.02  0.13  0.36  0.50 |

Average rate of exchange 2014-2016: US$1 = 559.8183 XOF

**Table S2 (continued).** Comparison of direct medical expenses (median and interquartile range) in USD by region between retrospective FFS and prospective FFS periods in Burkina Faso.

|  | Retrospective FFS period | Prospective FFS period | P-value |
| --- | --- | --- | --- |
| **Hauts Bassins**  Total expenses  Delivery fees  Drugs and supplies costs  Paraclinical exams costs  Hospitalization fees | **n=45**  6.02 (4.36-13.33)  1.34 (1.34-1.79)  3.61 (2.25-11.54)  0 (0-0)  0.89 (0.71-1.79) | **n=45**  6.36 (4.55-16.71)  1.34 (1.34-2.68)  3.70 (2.20-12.25)  0 (0-0)  0.89 (0.71-1.79) | 0.04  0.048  0.04  0.98  0.77 |
| **Centre-North**  Total expenses  Delivery fees  Drugs and supplies costs  Paraclinical exams costs  Hospitalization fees | **n=20**  7.37 (5.51-23.09)  1.25 (1.25-1.79)  5.22 (3.15-18.26)  0 (0-2.68)  0.89 (0.45-1.79) | **n=20**  8.95 (4.83-55.75)  1.30 (1.07-11.88)  7.16 (3.00-26.63)  0 (0-15.63)  0.89 (0.54-1.79) | 0.058  0.07  0.058  0.02  0.50 |
| **Centre-East**  Total expenses  Delivery fees  Drugs and supplies costs  Paraclinical exams costs  Hospitalization fees | **n=40**  8.17 (4.46-17.95)  1.34 (0.89-1.79)  6.34 (2.49-12.18)  0 (0-0)  0.89 (0.71-1.79) | **n=40**  10.42 (6.79-35.80)  2.86 (1.79-6.70)  6.95 (3.48-21.20)  0 (0-1.34)  1.79 (0.89-2.23) | <0.001  <0.001  <0.001  0.03  <0.001 |
| **East**  Total expenses  Delivery fees  Drugs and supplies costs  Paraclinical exams costs  Hospitalization fees | **n=25**  7.54 (5.18-29.66)  1.61 (1.34-6.43)  5.68 (3.43-19.65)  0 (0-3.57)  0.36 (0.36-0.89) | **n=25**  5.68 (4.11-24.63)  1.43 (1.34-6.43)  3.98 (2.27-16.42)  0 (0-3.57)  0.71 (0.36-1.79) | 0.66  0.29  0.79  0.09  0.32 |
| **North**  Total expenses  Delivery fees  Drugs and supplies costs  Paraclinical exams costs  Hospitalization fees | **n=28**  6.56 (4.33-41.58)  1.34 (1.34-1.88)  4.86 (2.63-24.29)  0 (0-12.06)  0.63 (0.36-2.23) | **n=28**  7.62 (4.43-70.96)  1.47 (1.34-13.58)  5.88 (2.73-34.84)  0 (0-16.08)  0.36 (0.36-4.47) | 0.02  <0.001  0.006  0.03  0.79 |
| **Sahel**  Total expenses  Delivery fees  Drugs and supplies costs  Paraclinical exams costs  Hospitalization fees | **n=23**  5.86 (3.91-12.78)  1.25 (0.89-2.95)  2.82 (2.30-8.99)  0 (0-0)  0.36 (0.36-1.07) | **n=23**  6.68 (5.39-16.69)  1.61 (1.34-2.23)  3.73 (2.82-13.12)  0 (0-0.06)  1.25 (0.89-1.79) | 0.001  0.13  0.005  0.02  0.02 |
| **Southwest**  Total expenses  Delivery fees  Drugs and supplies costs  Paraclinical exams costs  Hospitalization fees | **N=22**  5.20 (4.17-9.88)  1.34 (1.34-1.34)  3.16 (2.47-7.19)  0 (0-0)  0.45 (0.36-1.34) | **N=22**  8.15 (4.95-13.89)  1.34 (1.34-1.79)  5.24 (2.71-10.76)  0 (0-0)  1.79 (0.89-2.14) | 0.009  0.15  0.14  0.13  <0.001 |

Average rate of exchange 2014-2016: US$1 = 559.8183 XOF.
