# Supplementary material for: Effect of a prospective payment method for health facilities on direct medical expenditures in a low-resource setting: a paired pre-post study
Source: Health Policy Plan. 2020 Jun 4;35(7):775–83. doi: 10.1093/heapol/czaa039 (PMC7487330; doi:10.1093/heapol/czaa039)
Supplement: czaa039_supplementary_data [file czaa039_supplementary_data.zip › Table S3.doc]

**Table S3.** Average quantity and unit price of drugs and consumables used for delivery between the retrospective fee-for-service (FFS) and prospective FFS periods.

| Item | Retrospective FFS period | Prospective FFS period | p-value | Retrospective FFS period | Prospective FFS period | p-value |
| --- | --- | --- | --- | --- | --- | --- |
|  | Average number | |  | Average unit price (USD) | |  |
| Pair of gloves, sterile | 2.4 (2.0) | 3.0 (2.4) | <0.001 | 0.36 (0.04) | 0.36 (0.04) | 0.22 |
| Pair of gloves, nonsterile | 10.0 (9.0) | 15.9 (21) | <0.001 | 0.10 (0.02) | 0.10 (0.02) | 0.65 |
| Clamp de Barr | 0.8 (0.4) | 1.0 (0.3) | <0.001 | 0.16 (0.09) | 0.16 (0.10) | 0.99 |
| Syringes 10 CC | 2.0 (3.7) | 3.0 (7.0) | 0.02 | 0.12 (0.02) | 0.11 (0.01) | <0.001 |
| Syringes 5 CC | 0.6 (0.8) | 0.9 (1.7) | <0.001 | 0.09 (0.03) | 0.08 (0.01) | <0.001 |
| Injectable oxytocin (ampoule) | 2.8 (3.1) | 3.3 (3.8) | 0.08 | 0.30 (0.05) | 0.29 (0.01) | 0.02 |
| Injectable phloroglucinol | 0.5 (1.5) | 0.7 (1.8) | 0.03 | 0.78 (0.09) | 0.80 (0.09) | 0.37 |
| Compresses | 8.2 (15.6) | 9.7 (18.1) | 0.21 | 0.14 (0.02) | 0.14 (0.01) | 0.37 |
| Ringer lactate 500 ml | 0.6 (1.9) | 0.7 (1.9) | 0.50 | 1.17 (0.03) | 1.17 (0.04) | 0.54 |
| 5% glucose solution 500 ml | 0.5 (1.4) | 0.5 (1.5) | 0.73 | 1.21 (0.04) | 1.20 (0.02) | 0.01 |
| Solution of normal saline 500 ml | 0.4 (1.4) | 0.5 (1.3) | 0.32 | 1.13 (0.04) | 1.14 (0.04) | 0.55 |
| Intranule | 0.4 (0.8) | 0.5 (1.1) | 0.02 | 0.29 (0.07) | 0.27 (0.01) | 0.008 |
| Infusion sets | 0.3 (0.6) | 0.4 (0.7) | 0.02 | 0.35 (0.03) | 0.29 (0.06) | <0.001 |
| Surgical suture | 0.5 (1.2) | 0.7 (1.6) | 0.03 | 2.82 (0.89) | 3.01 (0.84) | 0.18 |
| Vitamin K1 (ampoule) | 0.2 (0.4) | 0.2 (0.5) | 0.097 | 0.36 (0.01) | 0.58 (1.00) | 0.08 |
| Amoxicillin 500 mg (blister pack) | 0.6 (1.2) | 0.8 (1.3) | 0.02 | 0.65 (0.09) | 0.63 (0.05) | 0.07 |

Average rate of exchange 2014-2016: US$1 = 559.8183 XOF.
